# Characterization of Spacesuit Associated Microbial Communities and Their Implications for NASA Missions

**DOI:** 10.3389/fmicb.2021.608478

**Published:** 2021-07-29

**Authors:** David Danko, Ganesh Babu Malli Mohan, Maria A. Sierra, Michelle Rucker, Nitin K. Singh, Aaron B. Regberg, Mary S. Bell, Niamh B. O’Hara, Rachid Ounit, Christopher E. Mason, Kasthuri Venkateswaran

**Affiliations:** ^1^Tri-Institutional Computational Biology & Medicine Program, Weill Cornell Medicine of Cornell University, Manhattan, NY, United States; ^2^The HRH Prince Alwaleed Bin Talal Bin Abdulaziz Alsaud Institute for Computational Biomedicine, Weill Cornell Medicine, New York, NY, United States; ^3^Biotechnology and Planetary Protection Group, Jet Propulsion Laboratory, California Institute of Technology, Pasadena, CA, United States; ^4^Department of Physiology and Biophysics, Weill Cornell Medicine, New York, NY, United States; ^5^Exploration Mission Planning Office, Johnson Space Center, Houston, TX, United States; ^6^Astromaterials Research and Exploration Science Division, Johnson Space Center, Houston, TX, United States; ^7^Jacobs@NASA/Johnson Space Center, Houston, TX, United States; ^8^Department of Computer Science and Engineering, University of California, Riverside, Riverside, CA, United States; ^9^The WorldQuant Initiative for Quantitative Prediction, Weill Cornell Medicine, New York, NY, United States; ^10^The Feil Family Brain and Mind Research Institute, Weill Cornell Medicine, New York, NY, United States

**Keywords:** spacesuit, microbial diversity, ISS, metagemonic, metagenome assembled genomes (MAGs), microbial ecology

## Abstract

**Background:**

Crewed National Aeronautics and Space Administration (NASA) missions to other solar system bodies are currently being planned. One high-profile scientific focus during such expeditions would be life detection, specifically the discovery of past or present microbial life, if they exist. However, both humans and associated objects typically carry a high microbial burden. Thus, it is essential to distinguish between microbes brought with the expedition and those present on the exploring planets. Modern spacesuits are unique, customized spacecraft which provide protection, mobility and life support to crew during spacewalks, yet they vent, and the mobility of microbes through spacesuits has not been studied.

**Results:**

To evaluate the microbial colonization of spacesuits, NASA used an Extravehicular Activity swab kit to examine viable microbial populations of 48 samples from spacesuits using both traditional microbiological methods and molecular sequencing methods. The cultivable microbial population ranged from below the detection limit to 9 × 10^2^ colony forming units per 25 cm^2^ of sample and also significantly varied by the location. The cultivable microbial diversity was dominated by members of *Bacillus, Arthrobacter*, and *Ascomycota.* However, 16S rRNA-based viable bacterial burden ranged from 10^5^ to 10^6^ copies per 25 cm^2^ of sample. Shotgun metagenome sequencing revealed the presence of a diverse microbial population on the spacesuit surfaces, including *Curtobacterium* and *Methylobacterium* from across all sets of spacesuits in high abundance. Among bacterial species identified, higher abundance of *Cutibacterium acnes*, *Methylobacterium oryzae*, and *M. phyllosphaerae* reads were documented.

**Conclusion:**

The results of this study provide evidence that identical microbial strains may live on the wrist joint, inner gauntlet, and outer gauntlet of spacesuits. This raises the possibility, but does not confirm that microbial contaminants on the outside of the suits could contaminate planetary science operations unless additional measures are taken. Overall, these data provide the first estimate of microbial distribution associated with spacesuit surfaces, which will help future mission planners develop effective planetary protection strategies.

## Introduction

Several spacefaring nations and private corporations are planning to send humans and spacecraft to other planets such as Mars, to search for evidence of habitats that could support life (NRC, 2014). Planetary Protection research efforts at National Aeronautics and Space Administration (NASA) seek to develop technologies to minimize any terrestrial microbial contamination to ensure the safety and health of astronauts, while also preserving scientific integrity of exoplanetary samples (NASA, 2019a). Planetary Protection aims involve the study and prevention of forward and back contamination, meaning the interchange of microbes and organic materials from Earth to other solar system bodies and vice versa (Debus and Arnould, 2008).

When astronauts will be sent to search for life on other planets, it will be necessary to understand what microorganisms they may bring with them. It is estimated that 85% of all microbial isolates recovered from spacecraft and supported facilities are microorganisms associated with the human microbiota (Nicholson et al., 2009). Accordingly, a team at the Johnson Space Center (JSC) at NASA has developed a prototype Extravehicular Activity (EVA) swab kit that is suitable for handling by the astronauts in spacesuits to collect microbial samples aseptically, aiming to profile microorganisms associated with spacesuits (Rucker et al., 2018). In this communication, a microbial characterization associated with wrist joints of flight Extravehicular Mobility Unit (EMU), Modified Advanced Crew Escape System and Orion Crew Survival System (MACES/OCSS) spacesuits was carried out to evaluate the form, fit and function of the EVA swab tool; that functional testing provided an opportunity to characterize the typical microbial contamination on spacesuits.

To explore and work in space, crew members must take their environment with them because there is no atmospheric pressure and no oxygen to sustain life. Inside the human crew vehicle, the atmosphere can be controlled so that special clothing is not necessary, but when outside exploring in space, astronauts need protection (Schwartz et al., 2002). Since various materials including fabrics and clothing are known to harbor specific microbiomes (Breuker et al., 2003; Cappitelli and Sorlini, 2008; Cataño et al., 2012; Callewaert et al., 2014; Sterndorff et al., 2020), it is of the highest interest to the NASA scientific community to explore the microbiome of the spacesuit (National Academies of Sciences, Engineering and Medicine, 2018). This study is not designed to understand the indigenous microbiome of the spacesuit when manufactured; instead spacesuit microbiome was measured when crew wear them after nominal handling and use to see how microorganisms might persist on the suits. Thorough characterization of spacesuit microbiome will enable the design of appropriate spacesuit architecture to minimize human commensal microorganism, which cannot be sterilized, from leaking into the external environment thus compromising life detection missions. Currently, all NASA spacesuits are designed to be flexible and which could lead to leakage. However, leak paths are not well-characterized, and it remains unclear what fraction of leakage occurs through mechanisms that would transport microbes. Characterization of spacesuits will also allow NASA to better understand cleaning process effectiveness for the spacesuits.

Since 2006, the field of genomics has been revolutionized by the development of next-generation sequencing technologies, enabling the comprehensive understanding of the microbial ecology of built environments such as offices (Chase et al., 2016), hospitals (Westwood et al., 2014), and transportation system environments (Hsu et al., 2016; Danko et al., 2021a) where humans spend a significant fraction of their time. Subsequently, molecular microbial community analyses were implemented to monitor the International Space Station (ISS) (Singh et al., 2018; Checinska Sielaff et al., 2019) and spacecraft assembly cleanrooms (Danko et al., 2021b) but this is the first report measuring spacesuit microbiome. While these technologies for microbial detection have elucidated the prevalence of microbial species, it was not until recently significant efforts have been pointed at developing sampling methods that enable sample collection in microgravity or a vacuum, that are simple to handle by crew members donned with large gloves, and that could preserve samples appropriately before performing subsequent molecular methods (Sandle, 2011; Rucker et al., 2018).

Since bulky EVA suits can restrict movement and limit visibility through the helmet visor, the primary objective was aimed to evaluate the interface between a fully suited test subject handling the EVA swab tool by the crew. Fully suited testing is important for identifying tool design issues prior to flight. At exploration destinations, such as Mars, suited crew may be required to periodically sample their suits as part of an environmental monitoring protocol. In addition, a benefit of this test was an opportunity to characterize the microorganisms found on or near selected suit pressure joints under vacuum and when the spacesuits were positively pressured, enabling NASA to assess exploration mission operations and hardware design to mitigate microbial leakage.

In this study EVA swab tools were used to collect several samples from variety of spacesuits (*n* = 7 sets; 48 samples) in a JSC training session. Spacesuit samples were treated with (allowing measurement of viable/intact cells) or without propidium monoazide (PMA, dead and alive cells) (48 samples each of PMA and no PMA; total *n* = 98 samples), a DNA intercalating dye before utilizing molecular technologies (Vaishampayan et al., 2013). The viable microbial burden targeting 16S rRNA gene (for bacteria/archaea) and internal transcribed region (ITS; for fungi) were estimated using quantitative polymerase chain reaction (qPCR) assay and shotgun metagenome sequencing (Singh et al., 2018). Furthermore, culturable microbial burden associated with spacesuits was measured using the traditional culture-based colony counts. This study will provide NASA with the ability to evaluate the spectrum of microbial diversity associated with spacesuits.

## Materials and Methods

### EVA Swab Material Selection

Validation of the macrofoam swabs (EVA swab tool material) to collect microorganisms from various material types was not a part of this study. However, a comprehensive study was performed previously to understand the suitable swab materials (cotton, polyester, and macrofoam) in the efficient removal of the microorganisms from the aluminum and titanium surfaces (Kwan et al., 2011). Briefly, a model microbial community comprised 11 distinct species of bacterial, archaeal, and fungal lineages, was used to examine the effects of variables in sampling matrices, target cell density/molecule concentration, and cryogenic storage on the overall efficacy of the sampling regimen. The biomolecules and cells/spores recovered from each collection device were assessed by cultivable and microscopic enumeration, and quantitative and species-specific PCR assays. rRNA gene-based quantitative PCR analysis showed that cotton swabs were superior to nylon-flocked swabs and macrofoam swabs significantly outperformed polyester wipes. Furthermore, macrofoam swab materials were found to withstand extreme temperature fluctuations of the space conditions including varying pressure, and vacuum (Rucker et al., 2018).

### EVA Swab Sample Kit Preparation and Sample Collection

**FIGURE 1:**
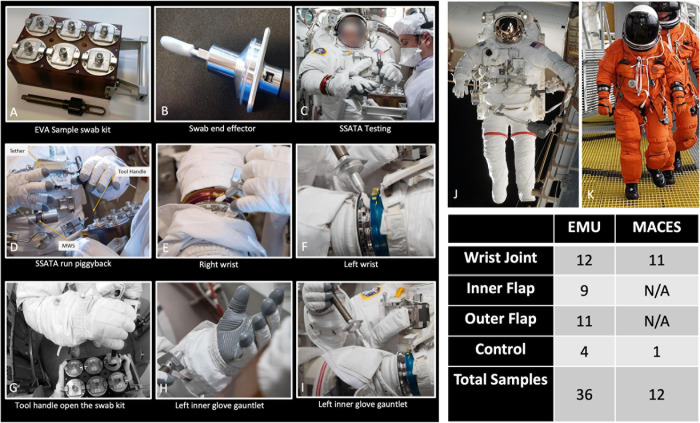
Photographs showing collection of various types of samples from different spacesuit types. **(A–I)** Images of sample collection and associated equipment, intended for use in flight. **(J)** An image of an Extravehicular Mobility Unit (EMU) suit. **(K)** An image of an Advanced Crew Escape Suit (ACES). Total number of samples collected are tabulated. N/A: Not available.

Three different kinds of spacesuits were sampled (Figure 1). Briefly, the EMU suits are currently used for EVA on ISS, but are not designed for use in planetary missions. We sampled stainless steel wrist joints and cloth gauntlets covering the joints on these suits. The outer fabric of the EMU is made of Ortho-Fabric, which is a blend of Gortex (ePTFE), Kevlar (a para-aramid type fiber related to nylon) and Nomex (a meta-aramid type fiber) (Newman et al., 2000). The MACES and OCSS suits designed for internal cabin use, such as inside Orion during launch and reentry through Earth’s atmosphere, use similar wrist joint as the EMU but without a gauntlet to cover it. The outer layer of the MACES and OCSS suits is comprised of orange Nomex (Watson, 2014; NASA, 2019b). NASA has conducted a series of ground tests intended to evaluate the EVA swab kit’s form, fit, and function under mission operations scenarios, in preparation for eventual sample collection from outside the ISS (Rucker et al., 2018). For samples collected from the EMU, EVA swabbing was an add-on to a routine suit familiarization test that all flight crew are required to perform. Familiarization involves suit fit and functional checks, followed by a 4-h prebreathe protocol (to mitigate potential for decompression sickness) before exposure to vacuum in the Space Station Airlock Test Article chamber. Spacesuit samples were collected during the prebreathe protocol, when the crew member was breathing pure oxygen at a suit internal pressure 4.3 psi higher than ambient external pressure, but not yet at external vacuum pressure. Although standard laboratory swabs could have been used under these conditions, this test provided an opportunity for suited crew to practice self-swabbing with the flight-like EVA swab kit, which will be necessary in future studies where samples will be taken under external vacuum conditions. A second series of tests was conducted with the MACES and OCSS suits. In these tests, four test subjects sampled their own suits (two MACES suits and two OCSS suits) inside the 11-foot vacuum chamber while the chamber was at vacuum (0.01 torr). The internal pressure inside the suits was 4.3 psi. Samples collected during these tests were exposed to a maximum of 4 h of vacuum.

Sample kit cleaning, sterilization, and assembly were performed at JSC according to a purpose-developed protocol. Each sample canister (assembled with filter and ball plungers) and swab end effector assembly was placed into separate autoclave bags. Bagged components were placed into Steris LV 250 laboratory steam sterilizer and sterilized using a gravity cycle of 45 min at 121°C (250°F) and 103.4 kPa (15 psi). Note that neither the sample caddy itself nor the tool handle which was never in contact with the swab head were autoclaved. Bagged components were allowed up to 1 h of cool-down time at approximately 22°C (71.6°F) for safe handling. Following autoclaving, bagged components were transferred to a Labconco Horizontal Clean Bench (Model # 36100000, ISO Class 5). With the commercial swab inside its sterile packaging, the swab stem was cut to optimal length [approximately 6.0 cm (2.4 in)] using sterilized wire cutters, making sure the swab head remained inside its packaging until the final assembly step. The cut end of the swab was then inserted into the end effector slot, and set screws were tightened to hold the swab in place. Sterile packaging was removed from the swab head immediately before inserting each swab assembly into its sterile container. Each container/swab assembly was then mounted into the tool caddy, wiped clean with isopropanol and placed into bonded storage until the test.

During swab assembly, technicians wore sterile gloves, and both the gloves and assembly tools (Allen wrench, scissors, and forceps) were sprayed with 70% ethanol surface disinfectant. All parts were handled either with sterile forceps or the autoclave bags, with no contact between the gloves and tool areas that must remain sterile. After assembly, the EVA sample kits were transported to the test site packed inside hard-sided storage cases. Once at the test site, the analog crew were briefed on tool usage and were given an opportunity to practice with a spare handle and sample caddy assembly. Over a period of 7 months between December 2016 and June 2017, 176 spacesuits, environmental control, and floor samples were collected during eight sampling time periods at JSC. Figure 1 shows sample collection from various parts of the spacesuits, EVA sampling kits, and number of samples associated with various spacesuits. The specific location for each sampling event of these 48 samples, surface area, and collection dates are given in Table 1 and detailed metadata about spacesuit types used, fabrics composition, microbial burden, cultivable diversity, are given in the Supplementary Tables 1, 2.

#### Controls

Among 48 spacesuit samples including five controls samples were further analyzed for various microbiological characteristics using traditional and shotgun metagenomic sequence analyses. Environmental controls were the swabs that were removed from the canister during testing but not touched to any surface. Negative controls were swabs that were not opened at all during testing. Among these 48 samples, 36 were from EMU and 12 were from MACES spacesuits.

### Sample Processing

After sample collection, sample processing took place in an ISO 7 (10K class) cleanroom at JPL. Under ISO 5 certified biosafety cabinet, each swab was aseptically severed with a sterile cutter and transferred to a 50 mL Falcon tube containing 15 mL of sterile phosphate-buffered saline (PBS; pH 7.4). The tube with the swab was shaken for 2 min followed by concentration with a Concentrating Pipette (Innova Prep, Drexel, MO, United States) using 0.22 μm Hollow Fiber Polysulfone tips (Cat #: CC08022) and PBS as elution fluid. Each sample was concentrated to 5 mL. A 100 μL concentrated aliquots were plated on various agar plates to estimate cultivable population using traditional plate count methods (described below). One mL of the diluted solution (200 μL plus 1.8 mL PBS) was used to conduct an ATP assay (Kikkoman Corp., Noda, Japan) to rapidly measure total and viable microbial population (Venkateswaran et al., 2003), enabling appropriate serial dilutions. Furthermore, 3 mL of each concentrated sample was split into two 1.5 mL- aliquots and one aliquot was treated with PMA to assess viability (Vaishampayan et al., 2013), while the second aliquot was handled similarly but without the addition of PMA. Briefly, 18.25 μL of 2 mM PMA was added to one half of the 3-mL sample (final concentration 25 μM) followed by 5 min incubation at room temperature in the dark and 15 min exposure to the activation system (PMA LED device, Biotium, Hayward, CA, United States). Each sample was then split into two 0.75 mL aliquots. One aliquot was transferred to bead beating tubes containing Lysing Matrix E (MP Biomedicals, Santa Ana, CA, United States), followed by bead beating for 60 s using the vortex sample holder (MO Bio, Carlsbad, CA, United States). The bead-beaten aliquot and the aliquot without bead beating were combined for their corresponding PMA-treated and non-treated samples. DNA extraction was accomplished with the Maxwell 16 automated system (Promega, Madison, WI, United States), in accordance with manufacturer instructions. A Maxwell control (MC) without any sample added in its cartridge was run concurrently with each flight sample set to account for microbial contamination associated with reagents (kitome) used in the automated DNA extraction. The extracted DNA was eluted in 50 μL of water and stored at −20°C and processed with the rest of the samples later.

### Estimation and Identification of Cultivable Microbial Population

The 100 μl of each concentrated sample were plated on Reasoner’s 2A agar (R2A for environmental microbes), Potato Dextrose Agar with chloramphenicol (100 μg/mL; PDA for fungi), and blood agar (BA for human commensals; Hardy Diagnostics, Santa Maria, CA, United States) in duplicate. R2A and PDA plates were incubated at 25°C for 7 days and BA plates at 37°C for 2 days at which time colony forming units (CFU) were counted. All colonies were picked from each plate and from each suit sampling location. The isolates were then archived in semisolid R2A or PDA slants (agar media diluted 1:10) and stored at room temperature. Once a culture was confirmed to be pure, two cryobead stocks (Copan Diagnostics, Murrieta, CA, United States) were prepared for each isolate and stored at –80°C. A loopful of purified microbial culture was directly subjected to PCR, and the targeted fragment was amplified (colony PCR), or DNA was extracted with the UltraClean DNA kit (MO Bio, Carlsbad, CA, United States) or Maxwell 16 instrument. The extracted DNA was used for PCR to amplify the 1.5 kb 16S rRNA gene to identify bacterial strains. The following primers were used for the 16S rRNA gene amplification to estimate bacterial population. The forward primer, 27F (5′-AGA GTT TGA TCC TGG CTC AG-3′) and the reverse primer, 1492R (5′-GGT TAC CTT GTT ACG ACT T-3′) (Lane, 1991; Turner et al., 1999). The PCR conditions were as follows: denaturation at 95°C for 5 min, followed by 35 cycles consisting of denaturation at 95°C for 50 s, annealing at 55°C for 50 s, and extension at 72°C for 1 min 30 s and finalized by extension at 72°C for 10 min. For fungal population estimation, the forward primer ITS 1F (5′-TTG GTC ATT TAG AGG AAG TAA-3′) (Lai et al., 2007) and reverse primer Tw13 (5′-GGT CCG TGT TTC AAG ACG-3′) (Taylor and Bruns, 1999) were used to obtain ∼1.2 kb ITS product. The PCR conditions were as follows: Initial denaturation at 95°C for 3 min followed by 25 cycles of 95°C for 50 s, annealing at 58°C for 30 s, and extension at 72°C for 2 min, followed by a final extension at 72°C for 10 min. The amplicons were inspected by gel electrophoresis in 1% agarose gel. When bands for products were visible, amplification products were treated with Antarctic phosphatase and exonuclease to remove 5′- and 3′- phosphates from unused dNTPs before sequencing. The sequencing was performed (Rockville, MD, United States) using 27F and 1492R primers for *Bacteria*, and ITS1F and Tw13 primers for fungi. The sequences were assembled using SeqMan Pro from DNAStar Lasergene Package (DNASTAR Inc., Madison, WI, United States). The bacterial sequences were searched against EzTaxon-e database (Kim et al., 2012) and the fungal sequences against the UNITE database (Koljalg et al., 2013). The identification was based on the closest percentage similarity (>97%) to previously identified microbial type strains.

### qPCR Assay

Following the DNA extraction, quantitative polymerase chain reaction (qPCR), targeting the partial 16S rRNA gene (bacteria) or partial ITS region (fungi), was performed with SmartCycler (Cepheid, CA, United States) to quantify the microbial burden as previously established (Checinska Sielaff et al., 2019). Each 25-μL reaction consisted of 12.5 μL of 2X iQ SYBR Green Supermix (BioRad, Hercules, CA, United States), 1 μL each of forward and reverse oligonucleotide primers (10 μM each), and 1 μL of template DNA (PMA treated and non-treated samples). Each sample was run in triplicate; the average and standard deviation were calculated based on these results. Purified DNA from a model microbial community (Kwan et al., 2011) served as the positive control and DNase/RNase free molecular-grade distilled water (Promega, Madison, WI, United States) was used as the negative control in each run. The number of gene copies was determined from the standard curve as described previously with a modification where synthetic fragments of *B. pumilus* (1.4 kb 16S rRNA gene) or *Aureobasidium pullulans* (1-kb ITS region) were used instead of genomic DNA (Checinska et al., 2015). The qPCR efficiency was ∼98%. The negative control values were not deducted since the values were at ∼100 copies per 1 or 10 μL and not scalable (yielded the same results despite using 1 μL and 10 μL of DNA templates).

#### Illumina Based DNA Sequencing and Analysis

The initial DNA yield and metagenome library quantitation of all 96 samples tested (48 samples PMA treated and 48 samples PMA untreated) were measured by Qbit (Thermo Fisher Scientific Inc., United States). DNA libraries for all 96 samples were prepared for shotgun metagenome sequencing using the Nextera DNA Library Preparation Kit from Illumina. The quality and fragment size of each library were assessed on a Bioanalyzer 2100 (Agilent). Separate adapters were added to the DNA from each library, normalized to 2 nM, pooled, denatured, and diluted to 1.8 pM according to the standard recommendations by Illumina. The HiSeq4000 platform (Illumina) was used for sequencing, resulting in 100-bp paired-end reads.

#### Bioinformatics Analysis

Bioinformatic analyses were performed on Weill Cornell Medicine’s Athena compute cluster, a typical high-performance grid compute (Slurm) system. The secondary analysis was performed on Linux and MacOS systems. Unless otherwise noted programs were run with default settings.

#### Data Quality Control and Filtering

Sequence data were processed with AdapterRemoval (v2.17) to remove low-quality reads and reads with ambiguous bases. Subsequently, reads were aligned to the human genome (hg38, including alternate contigs) using Bowtie2 (v2.3.0, fast preset). Read pairs where both ends mapped to the human genome were separated from read pairs where neither mate mapped. Read pairs where only one mate mapped were discarded. Hereafter, we refer to these read sets as human reads and non-human reads. We did not process human reads beyond counting the total fraction of DNA from our samples which mapped to the human genome.

#### Taxonomic Profiling and Analysis

Taxonomic profiles were generated by processing non-human reads KrakenUniq (v0.3.2) with a database based on all draft and reference genomes in RefSeq Microbial (bacteria, fungi, virus, and archaea) ca. March 2017. KrakenUniq uses a *k-*mer based approach to identify reads. Reads are broken into *k*-mers of 31 bases. Each *k*-mer is mapped to a database that lists the lowest common ancestor of all genomes which contained the *k*-mer. Each read is classified by identifying the best supported path in the taxonomic tree of markers. Finally, the taxonomic makeup of a sample is given by concatenating annotations for reads without further processing. KrakenUniq counts the number of unique marker *k*-mers assigned to each taxa, and we filtered taxa with fewer than 512 unique markers. Differential abundance estimation (where applicable) using the ALDEx2 R package was performed (Fernandes et al., 2013). Briefly, ALDEx2 transforms read count matrices using a centered log ratio transformation that models samples as Dirichlet-Multinomial distributions over taxa then compares taxonomic abundances across groups. If two groups are given, comparison is done with a Wilcoxon rank sum test, more than two groups are tested via a generalized linear model. All *p*-values are multiple hypotheses corrected using Benjamini-Hochberg. We considered a taxon to have differential abundance in a given condition if its corrected p-value was less than or equal to *p* = 0.05.

Dimensionality reduction of taxonomic profiles was performed with Uniform Manifold Approximation and Projection UMAP (McInnes et al., 2018) based on a matrix of Jensen-Shannon Divergences (JSD) between samples. Analysis of intersample diversity (beta-diversity) was achieved using the same matrix of JSD. Intrasample diversity (alpha-diversity) was measured by taking Shannon’s Entropy of the total sum normalized taxonomic profile of each sample. Rarefaction analysis of taxa was performed by grouping samples by location and setting and selecting 16 uniform random groups for each value. A curve of best fit was found by fitting a logarithmic model to the series.

Profile of Eukaryotic species were generated using CLARK-S (v1.2.5) (Ounit and Lonardi, 2016) using sequences classified with high confidence (i.e., confidence score > 0.75, and gamma score > 0.10) as defined in the CLARK manual. Identification of taxa was further restricted to species with relative abundance at least 0.01% of the total sequences.

Samples were compared to eight representative samples of human body sites selected from the Human Microbiome Project (HMP) (Turnbaugh et al., 2007) for each of five body sites: oral, skin, airways, gastrointestinal, and urogenital. Using MetaPhlAn2 (v2.2) (Truong et al., 2015), we generated taxonomic profiles for HMP samples and our samples and compared profiles using Cosine Similarity.

#### Functional Profiling and Analysis

HUMANn2 (Franzosa et al., 2018) was used to generate functional metabolic profiles of the genes in our samples. Non-human reads were aligned to Uniref90 (ca. March 2017) using the DIAMOND aligner (v0.8.6) (Buchfink et al., 2015). Subsequently, alignments were processed using HUMANn2 (v0.11.1) to produce profiles of pathway abundance. Pathways were tested for differential expression using the Wilcoxon rank sum corrected by Benjamini Hochberg. Dimensionality reduction of pathways was performed using PCoA over normalized pathway abundances.

#### Profiling Antimicrobial Resistance Genes

Profiles of antimicrobial resistance (AMR) genes using MegaRes (v1.0.1) (Lakin et al., 2017) were created. To generate profiles from MegaRes, we mapped non-human reads to the database using Bowtie2 (v2.3.0, very-sensitive presets) (Langmead and Salzberg, 2012). Subsequently, alignments were analyzed using ResistomeAnalyzer (commit 15a52dd) (Dean, 2018) and normalized by total reads per sample and gene length to give Reads per kilo base per million mapped reads (RPKMs). MegaRes includes an ontology grouping resistance genes into, gene classes, AMR mechanisms, and gene groups.

#### Identification of Genomes and Strains

We assembled contigs from all PMA treated samples using MegaHIT (v1.1.3) (Li et al., 2015) then clustered the resulting contigs into draft genomes using MetaBAT2 (Kang et al., 2019). Draft genomes were quality controlled and assigned a rough taxonomic rank using CheckM (Parks et al., 2015). Genomes with less than 50% completeness or more than 20% contamination were discarded. We aligned all genomes to one another to using Nucleotide MUMmer (Delcher et al., 2003) and processed the results to generate an Average Nucleotide Identity (ANI) between all pairs of draft genomes. We discarded all alignments that covered less than half the average lengths of the genomes being aligned. We further discarded alignments with less than 99% ANI so that we would only be left with pairs of nearly identical genomes. We grouped these alignments into connected components and analyzed the sites where each component was found.

## Results

### Microbial Abundance

A total of 48 samples (36 EMU and 12 MACES) were collected from six different surfaces of the spacesuits or environmental controls. Sampling surfaces include: left wrist joint (12 samples), left inner glove gauntlet (5 samples), left outer glove gauntlet (5 samples), right wrist joint (11 samples), right inner glove gauntlet (4 samples), right outer glove gauntlet (6 samples). All controls were analyzed for all microbiological and molecular biological examinations (5 samples, Table 1). All these 48 samples were categorized into sets (*n* = 7 sets) based on the suit types or sample collection dates (Table 1). In addition, metadata such as locations, type of suits, materials of spacesuits, and date of collection are given in Supplementary Table 1.

**TABLE 1:** Characteristics of various spacesuites sampled during this study and associated metadata.

**Set #**	**Sampled locations**	**Metagenome sample ID**	**Sampling date**	**Suit types sampled**	**Material type sampled**	**Sample pressure**	**PMA or no PMA**
SET-1	Control Swab – Not removed from canister	JC-044	12/28/2016	EMU	N/A	760	No PMA
SET-1	Control Swab – Not removed from canister	JC-092	12/28/2016	EMU	N/A	760	PMA
SET-1	Outside, left wrist gauntlet	JC-018	12/28/2016	EMU	Beta cloth	760	No PMA
SET-1	Outside, left wrist gauntlet	JC-066	12/28/2016	EMU	Beta cloth	760	PMA
SET-1	Inside, left wrist gauntlet	JC-013	12/28/2016	EMU	Beta cloth	760	No PMA
SET-1	Inside, left wrist gauntlet	JC-061	12/28/2016	EMU	Beta cloth	760	PMA
SET-1	Left wrist joint groove	JC-001	12/28/2016	EMU	Stainless Steel	760	No PMA
SET-1	Left wrist joint groove	JC-049	12/28/2016	EMU	Stainless Steel	760	PMA
SET-1	Outside, right wrist gauntlet	JC-038	12/28/2016	EMU	Beta cloth	760	No PMA
SET-1	Outside, right wrist gauntlet	JC-086	12/28/2016	EMU	Beta cloth	760	PMA
SET-1	Inside, right wrist gauntlet	JC-034	12/28/2016	EMU	Beta cloth	760	No PMA
SET-1	Inside, right wrist gauntlet	JC-082	12/28/2016	EMU	Beta cloth	760	PMA
SET-1	Right wrist joint groove	JC-023	12/28/2016	EMU	Stainless Steel	760	No PMA
SET-1	Right wrist joint groove	JC-071	12/28/2016	EMU	Stainless Steel	760	PMA
SET-2	Control Swab – Not removed from canister	JC-045	12/14/2016	EMU	N/A	760	No PMA
SET-2	Control Swab – Not removed from canister	JC-093	12/14/2016	EMU	N/A	760	PMA
SET-2	Outside, left wrist gauntlet	JC-019	12/14/2016	EMU	Beta cloth	760	No PMA
SET-2	Outside, left wrist gauntlet	JC-067	12/14/2016	EMU	Beta cloth	760	PMA
SET-2	Left wrist joint groove	JC-002	12/14/2016	EMU	Stainless Steel	760	No PMA
SET-2	Left wrist joint groove	JC-050	12/14/2016	EMU	Stainless Steel	760	PMA
SET-2	Outside, Right wrist gauntlet	JC-039	12/14/2016	EMU	Beta cloth	760	No PMA
SET-2	Outside, Right wrist gauntlet	JC-087	12/14/2016	EMU	Beta cloth	760	PMA
SET-2	Right wrist joint groove	JC-024	12/14/2016	EMU	Stainless Steel	760	No PMA
SET-2	Right wrist joint groove	JC-072	12/14/2016	EMU	Stainless Steel	760	PMA
SET-4	Long Term Control assembled 2/6 tested 3/16	JC-047	3/15/2017	EMU	N/A	760	No PMA
SET-4	Long Term Control assembled 2/6 tested 3/16	JC-095	3/15/2017	EMU	N/A	760	PMA
SET-3	Exterior, palm-side left wrist gauntlet	JC-020	2/6/2017	EMU	Beta cloth	760	No PMA
SET-3	Exterior, palm-side left wrist gauntlet	JC-068	2/6/2017	EMU	Beta cloth	760	PMA
SET-3	Left wrist joint groove	JC-003	2/6/2017	EMU	Stainless Steel	760	No PMA
SET-3	Left wrist joint groove	JC-051	2/6/2017	EMU	Stainless Steel	760	PMA
SET-3	Interior, left wrist gauntlet	JC-014	2/6/2017	EMU	Beta cloth	760	No PMA
SET-3	Interior, left wrist gauntlet	JC-062	2/6/2017	EMU	Beta cloth	760	PMA
SET-3	Right wrist joint groove	JC-025	2/6/2017	EMU	Stainless Steel	760	No PMA
SET-3	Right wrist joint groove	JC-073	2/6/2017	EMU	Stainless Steel	760	PMA
SET-3	Control Swab – Not removed from canister	JC-046	2/6/2017	EMU	N/A	760	No PMA
SET-3	Control Swab – Not removed from canister	JC-094	2/6/2017	EMU	N/A	760	PMA
SET-4	Left wrist outer gauntlet	JC-021	3/15/2017	EMU	Beta cloth	760	No PMA
SET-4	Left wrist outer gauntlet	JC-069	3/15/2017	EMU	Beta cloth	760	PMA
SET-4	Left wrist inner gauntlet	JC-015	3/15/2017	EMU	Beta cloth	760	No PMA
SET-4	Left wrist inner gauntlet	JC-063	3/15/2017	EMU	Beta cloth	760	PMA
SET-4	Left glove/lower arm groove	JC-004	3/15/2017	EMU	Stainless Steel	760	No PMA
SET-4	Left glove/lower arm groove	JC-052	3/15/2017	EMU	Stainless Steel	760	PMA
SET-4	Right wrist outer gauntlet	JC-040	3/15/2017	EMU	Beta cloth	760	No PMA
SET-4	Right wrist outer gauntlet	JC-088	3/15/2017	EMU	Beta cloth	760	PMA
SET-4	Right wrist inner gauntlet	JC-035	3/15/2017	EMU	Beta cloth	760	No PMA
SET-4	Right wrist inner gauntlet	JC-083	3/15/2017	EMU	Beta cloth	760	PMA
SET-4	Right glove/lower arm groove	JC-026	3/15/2017	EMU	Stainless Steel	760	No PMA
SET-4	Right glove/lower arm groove	JC-074	3/15/2017	EMU	Stainless Steel	760	PMA
SET-5	Control Swab – Not removed from canister	JC-048	3/16/2017	MACES/OCCS	N/A	0.01	No PMA
SET-5	Control Swab – Not removed from canister	JC-096	3/16/2017	MACES/OCCS	N/A	0.01	PMA
SET-5	Left wrist	JC-005	5/16/2017	OCCS	Stainless Steel	0.01	No PMA
SET-5	Left wrist	JC-053	5/16/2017	OCCS	Stainless Steel	0.01	PMA
SET-5	Right wrist	JC-027	5/16/2017	OCCS	Stainless Steel	0.01	No PMA
SET-5	Right wrist	JC-075	5/16/2017	OCCS	Stainless Steel	0.01	PMA
SET-5	Left wrist	JC-006	5/30/2017	MACES	Stainless Steel	0.01	No PMA
SET-5	Left wrist	JC-054	5/30/2017	MACES	Stainless Steel	0.01	PMA
SET-5	Right wrist	JC-028	5/30/2017	MACES	Stainless Steel	0.01	No PMA
SET-5	Right wrist	JC-076	5/30/2017	MACES	Stainless Steel	0.01	PMA
SET-6	Left wrist outer gauntlet	JC-022	5/30/2017	EMU	Beta cloth	760	No PMA
SET-6	Left wrist outer gauntlet	JC-070	5/30/2017	EMU	Beta cloth	760	PMA
SET-6	Left wrist inner gauntlet	JC-016	5/30/2017	EMU	Beta cloth	760	No PMA
SET-6	Left wrist inner gauntlet	JC-064	5/30/2017	EMU	Beta cloth	760	PMA
SET-6	Left glove/lower arm groove	JC-007	5/30/2017	EMU	Stainless Steel	760	No PMA
SET-6	Left glove/lower arm groove	JC-055	5/30/2017	EMU	Stainless Steel	760	PMA
SET-6	Right wrist outer gauntlet	JC-041	5/30/2017	EMU	Beta cloth	760	No PMA
SET-6	Right wrist outer gauntlet	JC-089	5/30/2017	EMU	Beta cloth	760	PMA
SET-6	Right wrist inner gauntlet	JC-036	5/30/2017	EMU	Beta cloth	760	No PMA
SET-6	Right wrist inner gauntlet	JC-084	5/30/2017	EMU	Beta cloth	760	PMA
SET-6	Right glove/lower arm groove	JC-029	5/30/2017	EMU	Stainless Steel	760	No PMA
SET-6	Right glove/lower arm groove	JC-077	5/30/2017	EMU	Stainless Steel	760	PMA
SET-6	Right wrist outer gauntlet	JC-042	6/14/2017	EMU	Beta cloth	760	No PMA
SET-6	Right wrist outer gauntlet	JC-090	6/14/2017	EMU	Beta cloth	760	PMA
SET-6	Left Wrist Crew 3	JC-008	6/8/2017	MACES	Stainless Steel	0.01	No PMA
SET-6	Left Wrist Crew 3	JC-056	6/8/2017	MACES	Stainless Steel	0.01	PMA
SET-6	Right Wrist Crew 3	JC-030	6/8/2017	MACES	Stainless Steel	0.01	No PMA
SET-6	Right Wrist Crew 3	JC-078	6/8/2017	MACES	Stainless Steel	0.01	PMA
SET-6	L Wrist Crew 4	JC-009	6/8/2017	MACES	Stainless Steel	0.01	No PMA
SET-6	L Wrist Crew 4	JC-057	6/8/2017	MACES	Stainless Steel	0.01	PMA
SET-6	Left wrist crew 3	JC-010	6/12/2017	OCCS	Stainless Steel	0.01	No PMA
SET-6	Left wrist crew 3	JC-058	6/12/2017	OCCS	Stainless Steel	0.01	PMA
SET-6	Right wrist crew 3	JC-031	6/12/2017	OCCS	Stainless Steel	0.01	No PMA
SET-6	Right wrist crew 3	JC-079	6/12/2017	OCCS	Stainless Steel	0.01	PMA
SET-6	Left wrist crew 4	JC-011	6/12/2017	MACES	Stainless Steel	0.01	No PMA
SET-6	Left wrist crew 4	JC-059	6/12/2017	MACES	Stainless Steel	0.01	PMA
SET-6	Right wrist crew 4	JC-032	6/12/2017	MACES	Stainless Steel	0.01	No PMA
SET-6	Right wrist crew 4	JC-080	6/12/2017	MACES	Stainless Steel	0.01	PMA
SET-7	Right wrist outer gauntlet	JC-043	6/26/2017	EMU	Beta cloth	760	No PMA
SET-7	Right wrist outer gauntlet	JC-091	6/26/2017	EMU	Beta cloth	760	PMA
SET-7	Right wrist inner gauntlet	JC-037	6/26/2017	EMU	Beta cloth	760	No PMA
SET-7	Right wrist inner gauntlet	JC-085	6/26/2017	EMU	Beta cloth	760	PMA
SET-7	Right glove/lower arm groove	JC-033	6/26/2017	EMU	Stainless Steel	760	No PMA
SET-7	Right glove/lower arm groove	JC-081	6/26/2017	EMU	Stainless Steel	760	PMA
SET-7	Left wrist inner gauntlet	JC-017	6/26/2017	EMU	Beta cloth	760	No PMA
SET-7	Left wrist inner gauntlet	JC-065	6/26/2017	EMU	Beta cloth	760	PMA
SET-7	Left glove/lower arm groove	JC-012	6/26/2017	EMU	Stainless Steel	760	No PMA
SET-7	Left glove/lower arm groove	JC-060	6/26/2017	EMU	Stainless Steel	760	PMA

*N/A, Not applicable; PMA, Propidium monoazide treated to measure viable microorganisms and no-PMA are the samples constitute both dead and live microbes; EMU, Extravehicular Mobility Unit; MACES, Modified Advanced Crew Escape System; OCCS, Onboard Complex Control System.*

**FIGURE 2:**
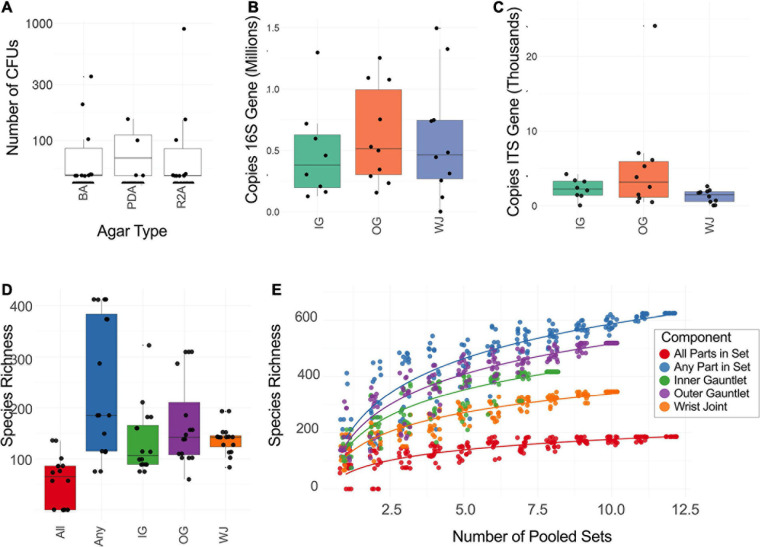
**(A)** Number of CFU found when plating samples on three different media. R2A, Reasoner’s 2A agar (for environmental bacteria); PDA, potato dextrose agar with chloramphenicol (100 μg/mL; for fungi); BA, blood agar (for human commensals). **(B)** Number of copies of the 16S rRNA gene found on different suit component: inner gauntlet (IG), outer gauntlet (OG), and wrist joint (WJ). **(C)** Number of copies of the ITS gene found on different components. **(D)** Total number of species detected by metagenomics on different components. *All* refers to microbes which were found in all components from a given set while *Any* refers to species on at least one component in a set. **(E)** Rarefaction curves showing the diversity of different numbers of components pooled together.

Our samples contained viable bacterial populations which were estimated by culture-dependent and independent analyses and are summarized in Supplementary Table 1 and Figure 2A. We cultured various microorganisms from our samples on three different types of media: blood agar, R2A, and PDA. The number of cultivable bacterial counts on R2A plates ranged from no growth to 9.0 × 10^2^ CFU per 25 cm^2^ (Figure 2A). The bacterial counts on blood agar were ranged from no growth to 3.5 × 10^2^ CFU per 25 cm^2^. No bacterial colonies were observed in any of the controls during this study. The phylogenetic affiliation of the bacterial strains isolated in this study was shown in Supplementary Figure 1A. Among 24 bacterial strains isolated and identified, the microorganisms belonged to the members of the phyla *Firmicutes* (13 strains), *Actinobacteria* (10 strains) and *Proteobacteria* (1 strain). *Bacillus* species represented the highest number of isolates, followed by *Arthrobacter* species. Comparatively, fungal isolates were not abundant and only six strains belonging to six different species were isolated. The ITS-based sequence analyses identified them as *Epicorum nigrum, Alternaria* sp., *Penicillium fagi, Aureobasidium pullulans, Naganishia adeliensis*, and *Neonectria* sp. The results of ATP-assay were not shown but ATP contents were used to estimate the microbial burden which further helped to determine appropriate serial dilutions.

A qPCR assay of the 16S and ITS genes was performed to measure the absolute microbial population of both viable (PMA treated) and total (PMA-untreated) microorganisms. This assay did not show a statistically significant difference in the microbial load among various locations sampled on spacesuits tested nor in various sets categorized. Viable bacterial load (PMA treated samples) was estimated at approximately 10^5^ to 10^6^ 16S rRNA copies per 25 cm^2^, Figure 2B. Viable bacterial population was an order of magnitude less abundant than total bacterial burden that include both dead and live microorganisms (Supplementary Table 1). Viable fungal population was measured at approximately 10^2^ to 10^4^ ITS copies per 25 cm^2^, Figure 2C. No significant difference was observed between EMU and MACES suits in either cultivable and culture-independent microbial burden assays.

### Molecular Microbial Diversity

Spacesuits are modular, each set refers to a single assembled set of components operated and sampled on a given day. The 48 samples including five controls were either treated with PMA or left untreated, resulting in an analysis of 96 samples. Among the 96 samples subjected for shotgun library preparation, all samples yielded enough DNA fragments except four PMA-treated samples and one non-PMA treated sample, hence 91 samples were subsequently assayed for shotgun metagenome sequencing. The PMA treated samples that did not yield any shotgun metagenome reads were SET-2 outside, left wrist gauntlet; SET-3 interior, left wrist gauntlet; SET-3 right wrist joint groove; and SET-7 left glove/lower arm groove.

In total, 319M reads were generated from all 91 samples. Human (∼38.2%) and animal (∼30%) associated reads were removed from the analyses. The PMA (49.8M) and non-PMA (54.7M) reads were ∼30% of the total reads. Approximately 104.5M reads associated with microorganisms were generated after high quality trimming from PMA (44 samples) and non-PMA treated (47 samples) samples. Dimensionality reduction comparing microbial taxonomic profiles between PMA treated and untreated samples showed an average shift based on PMA treatment (Supplementary Figure 2) suggesting that some types of microbes may be present on spacesuits as non-viable detritus. PMA treated samples were the focus of this study as they represent the intact/viable cells and information about PMA untreated samples were presented in supplementary datasets (Supplementary Tables 2, 3). The PMA-based analyses revealed that there were no microbial diversity differences among the EMU and MACES suits.

For all PMA treated samples, at domain level, the majority of the reads were assigned to bacteria (98.6%), followed by eukaryotes (0.9%), then archaea (0.24%), and viral signatures were 0.17%. For samples not treated with PMA, these reads were assigned to bacteria (98.6%), followed by eukaryotes (0.9%), archaea (0.5%), and viruses (0.1%). The proportional abundance of bacteria and fungi were similar in both PMA treated and non-PMA treated samples. When the relative abundance of all metagenomics reads was summed, ∼80% of the reads were attributed to the species whose reads were >100K.

None of the control samples yielded microbes that could be cultured in the media employed during this study which confirms that the EVA tool kit prepared for this study was sterile. But when all samples were considered for molecular analyses, ∼5% of the total metagenomics reads associated with bacteria, fungi, and viruses were present in control samples (*n* = 5). Among 993 microbial species observed in all spacesuits including control during this study (Supplementary Table 2), 13 bacterial taxa of control samples exhibited >10K reads and they were identified as *Bacillus pumilus, Cutibacterium acnes, Janthinobacterium* species (*n* = 3), *Micrococcus luteus, Negativicoccus massiliensis, Pseudomonas* species (*n* = 5), and *Ralstonia insidiosa.* Among them, *C. acnes, Janthinobacterium* species, *Pseudomonas* species, and *R. insidiosa* members were present in all five control samples. The bacterial species associated with controls that exhibited >100K reads were *N. massiliensis* (512K reads), *C. acnes* (448K reads), and *Pseudomonas* sp. NC02 (347K reads). Hence, few contaminant species were found as “kitomes” during this study and our finding is based on identifying microbial species/strains that are not in controls.

When various sets of spacesuits were compared, some differences were observed. Set #7 samples consist of members of the genera *Methylobacterium* and *Curtobacterium* whereas *Pseudomonas* species were prevalent in samples collected from set #5. Among 350 bacterial genera constituting 660 bacterial taxa identified, sequences of the members of the genera *Curtobacterium* and *Methylobacterium* were retrieved across all sets of spacesuits in high abundance. The compositional analysis showed a higher abundance of *Curtobacterium, Methylobacterium, Negativicoccus*, and *Pseudomonas* that exhibited more than two million reads. Among bacterial species identified (60 species > 100K reads; 239 species > 10K reads), higher abundance (>2M reads) of *Curtobacterium acnes* (8.9M reads), *Methylobacterium oryzae* (4.4M reads), and *M. phyllosphaerae* (4.2M reads) sequences were observed. Low fungal, archaeal, and viral reads were retrieved during this study and their sequence abundances and taxa characteristics are presented in Supplementary Tables 2–4.

### Molecular Microbial Diversity Indices

The total number of microbial species (species richness) found on each type of component (Inner Glove Gauntlet, Outer Glove Gauntlet, and Wrist Joint) was similar and typically between 100 and 200 (Figure 2C). A subset of these species could be found on all components in a set (typically 50–100 species found in all three components of either the left or right side of the suit) establishing a shared community. The inner and outer suit gauntlet had higher richness than the wrist joint (*p* < 2^–16^, one-way ANOVA).

To establish the total number of microbial species in the entire study (Figure 2D), a rarefaction analysis was performed (Figure 2E). Suits were considered as a whole and separately by component. A total of 660 microbial species were observed across all samples but a curve fit to the subsamples did not flatten which suggests that more microbial diversity would be seen with more samples collected. However, an analogous curve fit to subsets of species that occurred in all part in set did flatten, suggesting there may be a core community of 100–200 organisms common to spacesuits. Individual component types necessarily had more species than were found in all parts in set but fewer than were found in any part of a set.

To address the study design of collecting multiple samples from the same suit, we conducted a nested analysis using a regression Generalized Linear Mixed Model, and found that alpha diversity (Shannon Index) varied significantly across spacesuits for the PMA untreated group (*F*_5_,_35_ = 4.84, *P* = 0.002) but did not vary significantly for the PMA treated group. This may be due to the higher power demands of nested models and the limited number of samples collected.

### Taxonomic Analysis of Spacesuits

Microbial taxa were categorized based on a number of different conditions (1) differential abundance between PMA treated samples and untreated samples (determined by ALDEx2), (2) high prevalence taxa found in 31 out of 32 PMA treated EMU samples (excluding controls), (3) increased abundance in MACES suits compared to EMU suits for PMA treated samples (determined by ALDEx2), (4) differential abundance between suit components (wrist, inner, and outer gauntlets) in EMU samples treated with PMA (determined by ALDEx2). Differential abundance was defined as a Benjamini–Hochberg corrected *q*-value of 0.05 or less based on ALDEx2. Among PMA treated EMU samples one species *Corynebacterium kroppenstedtii* was identified as being significantly (*q* = 0.031) less abundant in wrist joint samples compared to other microbial species. Ninety-nine species were identified as differentially abundant in samples treated with PMA and untreated samples.

**FIGURE 3:**
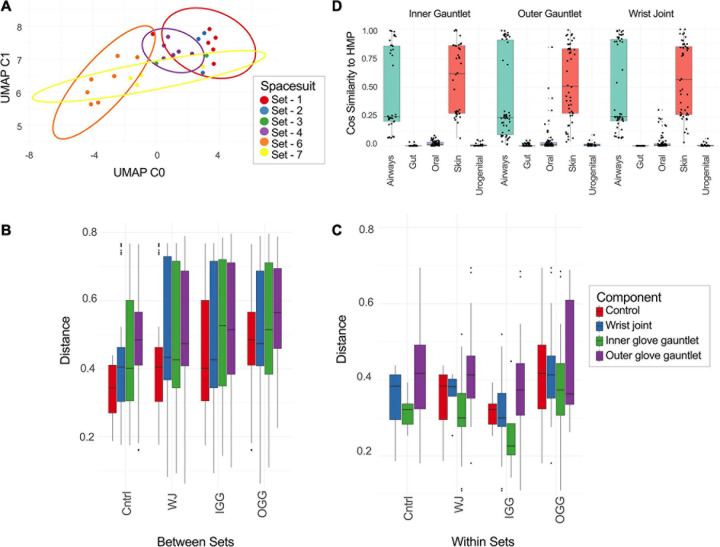
**(A)** UMAP of taxonomic profiles from PMA treated samples from EMU suits. Color indicates the set a sample came from. **(B,C)** Distance between different types of suit components [inner gauntlet (IG), outer gauntlet (OG), wrist joint (WJ), and controls (cntrl)] and samples from different sets **(B)** or the same set **(C)**. **(D)** The similarity of taxa from different components to representative samples from the Human Microbiome Project.

UMAP plot on the taxonomic profiles of samples (Supplementary Figure 2) and PMA treated samples only are depicted in Figure 3A. As expected, PMA treated sample clearly separated from untreated samples. This shows that there is a distinct likely viable set of microbes present on the sampled spacesuits. Within PMA treated samples, generally samples from the same suit clustered together with Sets 1 and 7 as notably tight clusters. Set 7 (all EMU suit sampled on June 26, 2017) was a definite outlier in relative abundance matching the pattern observed for alpha diversity.

### Beta Diversity and Sample Differentiation

The distance between taxonomic profiles of PMA treated samples of EMU suites was compared using JSD analysis. Dimensionality reduction of these distances using UMAP showed limited clustering by suit (Figure 3A). EMU suits were subdivided into eight triplets of samples that contained precisely one wrist, inner gauntlet, and outer gauntlet from the same suit. These triplets were in physical proximity to one another when sampled. We then compared two distributions: the distribution of distances between components in the same set and the distribution of distances between components in different sets (Figures 3B,C). The average JSD between components in the same set was 0.355 compared to 0.542 between components in different sets. A two-sided Welch’s *t*-test showed that these distributions did not share the same mean with *p*-value less than 2.0^–16^.

We also compared these distributions to distance distributions for control samples. The mean JSD between suit components and control samples collected at the same time was 0.365 while the mean distance between control samples and suit components from other sets was 0.470. The distance between components from different sets was larger than the difference between controls and components from other sets based on a two-sided Welch’s *t*-test with *p* of 1.01^–4^. Analogously, the distances between components from the same set were less than controls with a *p* of 2.17^–7^.

Taxonomic profiles of PMA treated samples during this study were compared to exemplar samples from the HMP. Spacesuit samples were found to be most similar to HMP skin and airway samples, suggesting that spacesuit microbiomes could originate from human skin or airway communities (Figure 3D). Notably the similarity to human body sites was not found to significantly vary by suit component or by which suit was being tested (one-way ANOVA), suggesting all components of all suits are exposed to human skin and airways microbiomes.

### Antimicrobial Resistance Genes

**FIGURE 4:**
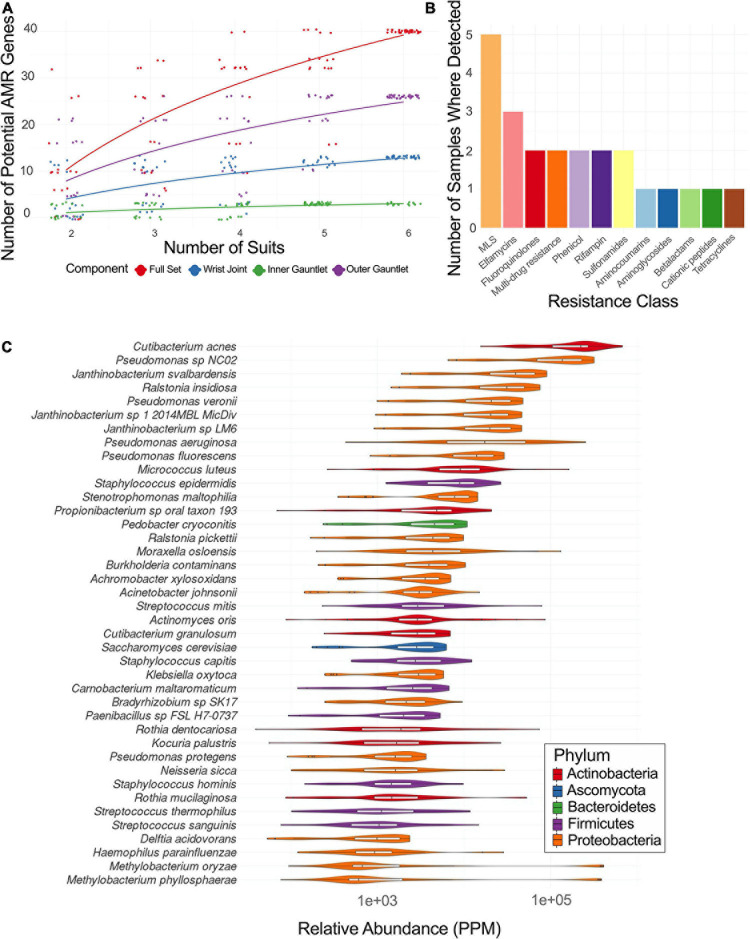
**(A)** Rarefaction plot of the number of AMR genes in different suit subsets. **(B)** Plot of the number of samples with AMR genes from a given resistance class. MLS stands for macrolide-lincosamide-streptogramin. **(C)** Relative abundance of major taxa in parts per million (PPM) across PMA treated samples from EMU suits.

Sequences of EMU PMA samples were mapped to known AMR genes and performed a rarefaction analysis of potential AMR genes (Figure 4A). Suits were considered as a whole and separately by component. Left and right gauntlet samples from the same component of the same suit were grouped together. A total observed richness of 40 AMR genes was noticed, but a curve fit to subsamples did not flatten, suggesting more diversity of AMR genes could be found. Samples from the outer gauntlet had more abundance than samples taken from the wrist, which in turn showed more AMR genes from the inner gauntlet. We grouped identified AMR genes by resistance class according to the MegaRes ontology. Five samples contained genes from the macrolide, lincosamide and streptogramin (MLS) class, 3 from the elfamycins, and just one sample contained a resistance gene from the beta-lactams (Figure 4B).

### Identified Microbial Species

Among the viable microbial species, *Pseudomonas* species were abundant in spacesuits studied. A core microbiome (occurring in 90% of samples or more) of 40 species in EMU samples treated with PMA was determined with several species in *Staphylococcus*, *Streptococcus, Pseudomonas*, and *Burkholderiales*. The distribution of abundances for microbial species with the highest median relative abundances was identified. *Cutibacterium acnes* was the most abundant taxa followed by several *Pseudomonas, Janthinobacterium*, and *Ralstonia insidiosa* (Figure 4C).

Fungal species (identified using CLARK-S, see methods) were also prevalent with 13 species identified in two or more samples. These include *Malassezia restricta* (found in all samples) which was associated with the skin microbiome of astronauts after their missions on the ISS by Sugita et al. (2016) and a number of other human commensal species. The full list of fungal species identified is given in Supplementary Table 3.

### Strain Specific Metagenome Assembled Genomes

**FIGURE 5:**
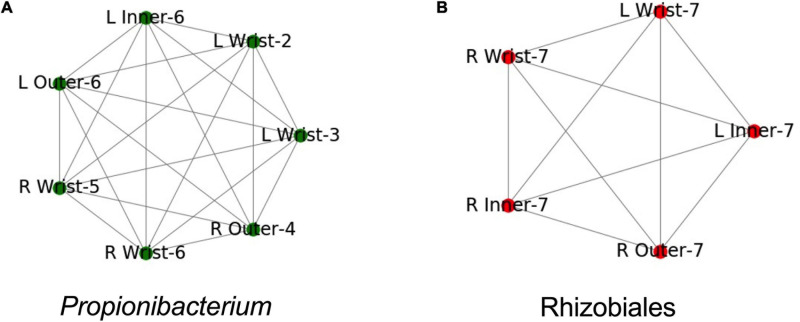
Both samples where identical taxa were assembled. Names are structured as Left (L) or Right (R), the suit component, and the set number. Edges indicate identical assemblies based on 99.5% ANI. **(A)** Samples where a *Propionibacterium* taxa was found. **(B)** Samples where a *Rhizobiales* taxa was found.

We built Metagenome Assembled Genomes (MAGs) from assemblies of all PMA treated samples, including controls. We identified MAGs that were found in more than one sample (99% ANI, see methods). These MAGs corresponded to two groups. One group of draft genomes was found in seven samples and was roughly categorized as a *Propionibacterium* species, the other group was found in five samples and was categorized as a member of *Rhizobiales* (Figures 5A,B). Both genome groups were fully connected, each draft genome from each sample had 99% ANI to each other sample in the group.

Both genomes were found in multiple samples from the same spacesuit. The *Propionibacterium* group was found in three samples from Set 6: the right wrist, and the inner and outer left gauntlets (Figure 5A). The *Rhizobiales* group was found in five samples from Set 7: the right wrist, inner and outer gauntlets and the left wrist and inner gauntlet (Figure 5B). Neither genome was found in any control sample. Since the samples where the genomes were found were treated with PMA, we further conclude that these microorganisms were likely viable.

The incidence of multiple examples of the same genome at wrist, inner, and outer gauntlets of two suits is consistent with the possibility that viable bacteria might have migrated from the inside to the outside of a spacesuit, but could also have been deposited in all three locations during the suit donning process. For crew health, surfaces inside the space suits were cleaned using stericide wipes; however exterior surfaces (including the surfaces that were sampled), were not cleaned. The stericide wipes chemical components were: 1.5% *n*-alkyl dimethyl benzyl ammonium chloride (60% C_14_, 30% C_16_, 5% C_12_, 5% C_18_) and 1.5% *n*-alkyl ethylbenzyl ammonium chloride (50% C_12_, 30% C_14_, 17% C_16_, 5% C_18_) as maintenance procedure. Since neither MAG was found in any control it is concluded that presence of these genomes is not due to contamination but might be due to migration from one location to another location of the spacesuit.

## Discussion

In this study, we established that viable microbes (and their MAGs) are present on the wrist assemblages of spacesuits, that certain microbial strains can survive on all three components of the wrist assembly without being found in corresponding controls, and that these microbes broadly resemble those of human commensal skin microbiomes.

Microorganisms associated with fabrics have not historically been studied in detail (Buschle-Diller et al., 1994; Cappitelli and Sorlini, 2008; Linacre et al., 2010; Daly et al., 2012; Lee et al., 2016), whereas microbiome of human (Turnbaugh et al., 2007; Nelson et al., 2010; Jensen, 2013; Shafquat et al., 2014) and built environments (Danko et al., 2021a), including closed habitat of ISS environment (Singh et al., 2018; Checinska Sielaff et al., 2019), has received much attention (Westwood et al., 2014; Kettleson et al., 2015; Chase et al., 2016; Lax et al., 2017). One of the objectives of this study was determining if a human within a spacesuit could act as a source for the unintentional microbial contamination and pass on microbial signatures out of the spacesuits. Future in-depth sampling and testing of various spacesuit components are necessary to conclude the transmission route. A detailed microbiome analyses of spacesuit before crew occupation should be carried out and such samples are not available for this study.

Microorganisms might escape through the clothing fibers via adherence, growth, and damage to the fibers. When synthetic fabrics were evaluated for microbial composition, micrococci were predominantly isolated both via culture and molecular methods (Callewaert et al., 2014). Prior studies have also revealed abundance of staphylococci on both cotton and synthetic fabrics, but corynebacteria were not enriched on any textile types, indicating that clothing fiber composition might promote differential growth of distinct microbes. Similarly, the spacesuits examined during the study revealed isolation of actinobacteria and staphylococci, but members of corynebacteria were not isolated using traditional methods (Supplementary Figure 1A). However, the culture-independent analyses showed presence of corynebacteria (Figure 4C).

Characterization of microorganisms degrading the synthetic polymers such as polyvinyl chloride (PVC), polyurethane, nylon, and acrylics and their mode of action have been reviewed (Cappitelli and Sorlini, 2008). As shown in this study black fungi were isolated (Supplementary Figure 1B), however colonization of PVCs by these fungi and their degrading capabilities of plasticizers should be assessed before their concluding potential polymer degradation (Roberts and Davidson, 1986; Webb et al., 2000). The sequences associated with *Candida albicans*, a common skin microbiota, were not retrieved during this study, but retrieval of sequence from taxonomically related *Candida dubliniensis* was found from the majority of suit samples and not from controls.

The composition of the fabrics and particles associated with them might determine the interaction of the microbes and fiber, but such phenomenon is not always uniform and large discrepancies exist. Fabric materials that are plant-based (e.g., cotton) might provide nutrients for microorganisms to degrade and also cotton fabrics were reported to adsorb sweat components thus promotes growth of microorganisms (Szostak-Kotowa, 2004). Several enzymes were reported to degrade fibers like cellulose and notably fungi secreting cellulolytic enzymes (Buschle-Diller et al., 1994). Even though synthetic fibers last longer than natural fabrics like cotton, they collect moisture between the fibers and become nutrients source for microorganisms (Szostak-Kotowa, 2004). However, during this study, no differences in microbial burden was noticed when EMU and MACES/OCCS suits were compared.

When all PMA-treated samples were pooled into various locations of the spacesuit such as wrist joint (*n* = 23), inner gauntlet (*n* = 10), and outer gauntlet (*n* = 10), opportunistic pathogens were found to be associated with wrist joint rather than the inner or outer gauntlet samples (Table 2). Notably, *C. kroppenstedtii*, an opportunistic pathogen, has a high relative abundance in wrist joints (∼10K reads) compared to inner gauntlets (∼1K reads) and outer gauntlets (630 reads). *Rothia dentocariosa*, an oral cavity microbe, was found more in outer gauntlet. Similarly, the microbiome of outer and inner gauntlets appeared to harbor microorganisms associated with soil (*Pseudomonas stutzeri*) as well as the radiation-resistant *Methylobacterium radiotolerans.* The possibility of microbes surviving harsh conditions associated with space by migrating on or through spacesuits should be explored with controlled experiment before drawing the movement of microbes from one location to another location.

**TABLE 2:** Potential transmission of viable microorganisms among various locations of space suits.

**Taxa**	Number of PMA reads* retrieved from:		
	**Wrist joint (n=23)**	**Inner gauntlet (n=10)**	**Outer gauntlet (n=10)**
*Acinetobacter baumannii*	1,364	1,088	
*Enterobacter cloacae*	402	736	
*Corynebacterium kroppenstedtii*	9,987	1,102	630
*Rothia dentocariosa*	1,132	804	6,972
*Kocuria rhizophila*	1,022	7,258	534
*Pseudomonas stutzeri*	1,917	763	214,233
*Methylobacterium radiotolerans*	102,052	214,816	110,274
*Methylobacterium extorquens*	12,163	26,274	12,994
*Methylobacterium nodulans*	1,245	2,710	1,335
*Methylobacterium populi*	2,274	4,842	2,413
*Methylobacterium* sp 4-46	2,221	4,709	2,306
*Streptococcus thermophilus*	2,200	1,027	625

*The shotgun metagenome reads generated after PMA treatment andretrieved from various locations were pooled to generate potential transmission of the viable microorganisms from one location to another.

Detecting microbes that were reported to be potentially harmful to astronaut health might be a concern. Members of *Methylobacterium* (12 species) dominated microbial communities on set #7 suits in this study, were reported to be opportunistic pathogens and might cause infections to immunocompromised patients (Kovaleva et al., 2014). Moreover, since astronauts are in close contact with suits while in use and shared suits present a hypothetical route for pathogen transmission, these measures can help inform potential risk. Though our work was limited to the exterior of suits, this study documented that spacesuit could harbor viable microbes. None of the microbes discovered are thought to present a health risk, but some belong to clades that contain potential pathogens. All of them represent organisms that may be relevant for NASA planetary protection (NASA, 2019a), since they may persist on the suit.

## Conclusion

The microbial characterization of spacesuits examined during this study established the following scenarios. (1) Viable microbes are present on the exterior of spacesuits. (2) Certain microbes exist on suit joints and gauntlets. (3) The microbiomes on suit surfaces resemble those of human skin and airways. More sophisticated approaches can help to conclusively establish whether microbes do migrate from the interior to the exterior of pressurized suits in space.

Additional work to better determine microbial origin and evaluate microbial contamination mitigation techniques is warranted. This report is a first step in establishing a catalog of microbial sequences known to occur on spacesuits and equipment. Gene specific marker, single nucleotide polymorphism (gene property), single nucleotide variation, and with deep coverage (×100) should be performed to track the source of microbial passage between the exterior and interior of currently existing spacesuits.

## Data Availability Statement

The datasets presented in this study can be found in online repositories. The names of the repository/repositories and accession number(s) can be found below: https://www.ncbi.nlm.nih.gov/genbank/, PRJNA545796.

## Author Contributions

MR developed EVA tool, collected samples, coordinated and designed the study with input from KV. MR, MB, and AR collected samples at JSC. MB and AR wrote the cleaning, sterilization, and assembly protocol for the EVA Swab Kit. GM contributed to sample processing, traditional microbiology assays, extracted DNA, assayed molecular microbial burden, generated corresponding figures, tables, and text associated with these analyses. CM group generated shotgun metagenome sequencing. DD designed, coordinated, and carried out computational analyses. NO’H and RO assisted with strain level analyses. NS conducted metagenome-based phylogenetic and functional analyses and interpreted the results. KV, DD, and MS drafted the manuscript and responsible for data analysis and interpretation. CM edited the manuscript. All authors read and approved the final manuscript.

## Conflict of Interest

The authors declare that the research was conducted in the absence of any commercial or financial relationships that could be construed as a potential conflict of interest.

## Publisher’s Note

All claims expressed in this article are solely those of the authors and do not necessarily represent those of their affiliated organizations, or those of the publisher, the editors and the reviewers. Any product that may be evaluated in this article, or claim that may be made by its manufacturer, is not guaranteed or endorsed by the publisher.

## Supplementary Material

Supplementary Figure 1 Phylogenetic tree of cultivated strains (bacteria **[A]** and fungi **[B]**) isolated from spacesuit surfaces.

Supplementary Figure 2 UMAP of taxonomic profiles from all samples colored by whether the samples were treated with PMA or not.

Supplementary Table 1 Metadata for samples including what suit they were taken from and when.

Supplementary Table 2 Bacterial abundances (in number of reads) from metagenomics for all samples.

Supplementary Table 3 Eukaryote abundances (in number of reads) from metagenomics for all samples.

Supplementary Table 4 Percentage of reads assigned to different major groups.

## References

[B1] Breuker M. McNamara C. Young L. Perry T. Young A. Mitchell R. (2003). Fungal growth on synthetic cloth from Apollo spacesuits. *Ann. Microbiol.* 53 47–54.

[B2] Buchfink B. Xie C. Huson D. H. (2015). Fast and sensitive protein alignment using DIAMOND. *Nat. Methods* 12 59–60. 10.1038/nmeth.3176 25402007

[B3] Buschle-Diller G. Zeronian S. H. Pan N. Yoon M. Y. (1994). Enzymatic hydrolysis of cotton, linen, ramie, and viscose rayon fabrics. *Text. Res. J.* 64 270–279. 10.1177/004051759406400504

[B4] Callewaert C. De Maeseneire E. Kerckhof F.-M. Verliefde A. Van de Wiele T. Boon N. (2014). Microbial odor profile of polyester and cotton clothes after a fitness session. *Appl. Environ. Microbiol.* 80 6611–6619. 10.1128/AEM.01422-14 25128346 PMC4249026

[B5] Cappitelli F. Sorlini C. (2008). Microorganisms attack synthetic polymers in items representing our cultural heritage. *Appl. Environ. Microbiol.* 74 564–569. 10.1128/aem.01768-07 18065627 PMC2227722

[B6] Cataño J. C. Echeverri L. M. Szela C. (2012). Bacterial contamination of clothes and environmental items in a third-level hospital in Colombia. *Interdiscipl. Perspect. Infect. Dis.* 2012:507640. 10.1155/2012/507640 22536231 PMC3321286

[B7] Chase J. Fouquier J. Zare M. Sonderegger D. L. Knight R. Kelley S. T. (2016). Geography and location are the primary drivers of office microbiome composition. *mSystems* 1:e0022-16. 10.1128/mSystems.00022-16 27822521 PMC5069741

[B8] Checinska A. Probst A. J. Vaishampayan P. White J. R. Kumar D. Stepanov V. G. (2015). Microbiomes of the dust particles collected from the international space station and spacecraft assembly facilities. *Microbiome* 3:50. 10.1186/s40168-015-0116-3 26502721 PMC4624184

[B9] Checinska Sielaff A. Urbaniak C. Mohan G. B. M. Stepanov V. G. Tran Q. Wood J. M. (2019). Characterization of the total and viable bacterial and fungal communities associated with the International Space Station surfaces. *Microbiome* 7:50. 10.1186/s40168-019-0666-x 30955503 PMC6452512

[B10] Daly D. J. Murphy C. McDermott S. D. (2012). The transfer of touch DNA from hands to glass, fabric and wood. *Forens. Sci. Int. Genet.* 6 41–46. 10.1016/j.fsigen.2010.12.016 21330229

[B11] Danko D. Bezdan D. Afshin E. E. Ahsanuddin S. Bhattacharya C. Butler D. J. (2021a). A global metagenomic map of urban microbiomes and antimicrobial resistance. *Cell* 184 3376–3393e17. 10.1016/j.cell.2021.05.002 34043940 PMC8238498

[B12] Danko D. C. Sierra M. A. Benardini J. N. Guan L. Wood J. M. Singh N. (2021b). A comprehensive metagenomics framework to characterize organisms relevant for planetary protection. *Microbiome* 9 1–15. 33795001 10.1186/s40168-021-01020-1 PMC8016160

[B13] Dean C. (2018). *Resistome Analyzer.* Available online at: https://github.com/cdeanj/resistomeanalyzer

[B14] Debus A. Arnould J. (2008). Planetary protection issues related to human missions to Mars. *Adv. Space Res.* 42 1120–1127. 10.1016/j.asr.2007.10.005

[B15] Delcher A. L. Salzberg S. L. Phillippy A. M. (2003). Using MUMmer to identify similar regions in large sequence sets. *Curr. Protoc. Bioinform.* 10:Unit10.3. 10.1002/0471250953.bi1003s00 18428693

[B16] Fernandes A. D. Macklaim J. M. Linn T. G. Reid G. Gloor G. B. (2013). ANOVA-like differential expression (ALDEx) analysis for mixed population RNA-Seq. *PLoS One* 8:e67019. 10.1371/journal.pone.0067019 23843979 PMC3699591

[B17] Franzosa E. A. McIver L. J. Rahnavard G. Thompson L. R. Schirmer M. Weingart G. (2018). Species-level functional profiling of metagenomes and metatranscriptomes. *Nat. Methods* 15 962–968. 10.1038/s41592-018-0176-y 30377376 PMC6235447

[B18] Hsu T. Joice R. Vallarino J. Abu-Ali G. Hartmann E. M. Shafquat A. (2016). Urban transit system microbial communities differ by surface type and interaction with humans and the environment. *mSystems* 1:e0018-16. 10.1128/mSystems.00018-16 27822528 PMC5069760

[B19] Jensen G. L. (2013). *The Human Microbiome, Diet, and Health: Workshop Summary.* Washington, DC: National Academies Press. 23967499

[B20] Kang D. D. Li F. Kirton E. Thomas A. Egan R. An H. (2019). MetaBAT 2: an adaptive binning algorithm for robust and efficient genome reconstruction from metagenome assemblies. *PeerJ* 7:e7359. 10.7717/peerj.7359 31388474 PMC6662567

[B21] Kettleson E. M. Adhikari A. Vesper S. Coombs K. Indugula R. Reponen T. (2015). Key determinants of the fungal and bacterial microbiomes in homes. *Environ. Res.* 138 130–135. 10.1016/j.envres.2015.02.003 25707017 PMC4385485

[B22] Kim O.-S. Cho Y.-J. Lee K. Yoon S.-H. Kim M. Na H. (2012). Introducing EzTaxon-e: a prokaryotic 16S rRNA gene sequence database with phylotypes that represent uncultured species. *Intern. J. Syst. Evol. Microbiol.* 62 716–721. 10.1099/ijs.0.038075-0 22140171

[B23] Koljalg U. Nilsson R. H. Abarenkov K. Tedersoo L. Taylor A. F. Bahram M. (2013). Towards a unified paradigm for sequence-based identification of fungi. *Mol. Ecol.* 22 5271–5277. 10.1111/mec.12481 24112409

[B24] Kovaleva J. Degener J. E. van der Mei H. C. (2014). *Methylobacterium* and its role in health care-associated infection. *J. Clin. Microbiol.* 52 1317–1321. 10.1128/jcm.03561-13 24430456 PMC3993692

[B25] Kwan K. Cooper M. La Duc M. T. Vaishampayan P. Stam C. Benardini J. N. (2011). Evaluation of procedures for the collection, processing, and analysis of biomolecules from low-biomass surfaces. *Appl. Environ. Microbiol.* 77 2943–2953. 10.1128/aem.02978-10 21398492 PMC3126404

[B26] Lai X. Cao L. Tan H. Fang S. Huang Y. Zhou S. (2007). Fungal communities from methane hydrate-bearing deep-sea marine sediments in South China See. *ISME J.* 1 756–762. 10.1038/ismej.2007.51 18059498

[B27] Lakin S. M. Dean C. Noyes N. R. Dettenwanger A. Ross A. S. Doster E. (2017). MEGARes: an antimicrobial resistance database for high throughput sequencing. *Nucleic Acids Res.* 45 D574–D580. 27899569 10.1093/nar/gkw1009 PMC5210519

[B28] Lane D. J. (1991). “Nucleic acid techniques in bacterial systematics,” in *Nucleic Acid Techniques in Bacterial Systematics*, Vol. 1 eds Stackebrandt E. Goodfellow M. (New York, NY: Wiley), 115–175.

[B29] Langmead B. Salzberg S. L. (2012). Fast gapped-read alignment with Bowtie 2. *Nat. Methods* 9:357. 10.1038/nmeth.1923 22388286 PMC3322381

[B30] Lax S. Sangwan N. Smith D. Larsen P. Handley K. M. Richardson M. (2017). Bacterial colonization and succession in a newly opened hospital. *Sci. Transl. Med.* 9:eaah6500. 10.1126/scitranslmed.aah6500 28539477 PMC5706123

[B31] Lee S.-Y. Woo S.-K. Lee S.-M. Eom Y.-B. (2016). Forensic analysis using microbial community between skin bacteria and fabrics. *Toxicol. Environ. Health Sci.* 8 263–270. 10.1007/s13530-016-0284-y

[B32] Li D. Liu C.-M. Luo R. Sadakane K. Lam T.-W. (2015). MEGAHIT: an ultra-fast single-node solution for large and complex metagenomics assembly via succinct de Bruijn graph. *Bioinformatics* 31 1674–1676. 10.1093/bioinformatics/btv033 25609793

[B33] Linacre A. Pekarek V. Swaran Y. C. Tobe S. S. (2010). Generation of DNA profiles from fabrics without DNA extraction. *Forens. Sci. Int. Genet.* 4 137–141. 10.1016/j.fsigen.2009.07.006 20129473

[B34] McInnes L. Healy J. Melville J. (2018). Umap: Uniform manifold approximation and projection for dimension reduction. *arXiv* [Preprint]. Available online at: https://arxiv.org/abs/1802.03426

[B35] NASA (2019a). *NASA Policy Instruction-8020.7G: NASA Policy on Planetary Protection Requirements for Human Extraterrestrial Missions.* Washington, DC: NASA.

[B36] NASA (2019b). *Orion Suit Equipped to Expect the Unexpected on Artemis Missions.* Washington, DC: NASA.

[B37] National Academies of Sciences, Engineering and Medicine (2018). *A Midterm Assessment of Implementation of the Decadal Survey on Life and Physical Sciences Research at NASA.* Washington, DC: The National Academies Press. 29924532

[B38] Nelson K. E. Weinstock G. M. Highlander S. K. Worley K. C. Creasy H. H. Wortman J. R. (2010). A catalog of reference genomes from the human microbiome. *Science* 328 994–999. 20489017 10.1126/science.1183605 PMC2940224

[B39] Newman D. Schmidt P. Rahn D. (2000). *Modeling the Extravehicular Mobility Unit (EMU) Space Suit: Physiological Implications for Extravehicular Activity (EVA) (0148-7191).* Available online at: http://web.mit.edu/aeroastro/www/people/dnewman/pdfs/DJN_ICES2000-3.26.pdf

[B40] Nicholson W. L. Schuerger A. C. Race M. S. (2009). Migrating microbes and planetary protection. *Trends Microbiol.* 17 389–392. 10.1016/j.tim.2009.07.001 19726193

[B41] NRC (2014). *Pathways to Exploration: Rationales and Approaches for a U.S. Program of Human Space Exploration.* Washington, DC: The National Academies Press.

[B42] Ounit R. Lonardi S. (2016). Higher classification sensitivity of short metagenomic reads with CLARK-S. *Bioinformatics* 32 3823–3825. 10.1093/bioinformatics/btw542 27540266

[B43] Parks D. H. Imelfort M. Skennerton C. T. Hugenholtz P. Tyson G. W. (2015). CheckM: assessing the quality of microbial genomes recovered from isolates, single cells, and metagenomes. *Genome Res.* 25 1043–1055. 10.1101/gr.186072.114 25977477 PMC4484387

[B44] Roberts W. T. Davidson P. M. (1986). Growth characteristics of selected fungi on polyvinyl chloride film. *Appl. Environ. Microbiol.* 51 673–676. 10.1128/aem.51.4.673-676.1986 3707118 PMC238945

[B45] Rucker M. A. Hood D. Walker M. Venkateswaran K. J. Schuerger A. C. (2018). EVA swab tool to support planetary protection and astrobiology evaluations. *Paper Presented at the 2018 IEEE Aerospace Conference*, New York, NY.

[B46] Sandle T. (2011). A review of cleanroom microflora: types, trends, and patterns. *PDA J. Pharm. Sci. Technol.* 65 392–403. 10.5731/pdajpst.2011.00765 22293526

[B47] Schwartz S. J. Hoffman J. A. Hodgson E. Ronzani P. A. (2002). Is there space for wearables?. *Paper Presented at the Proceedings of the Sixth International Symposium on Wearable Computers*, London.

[B48] Shafquat A. Joice R. Simmons S. L. Huttenhower C. (2014). Functional and phylogenetic assembly of microbial communities in the human microbiome. *Trends Microbiol.* 22 261–266. 10.1016/j.tim.2014.01.011 24618403 PMC4008634

[B49] Singh N. K. Wood J. M. Karouia F. Venkateswaran K. (2018). Succession and persistence of microbial communities and antimicrobial resistance genes associated with International Space Station environmental surfaces. *Microbiome* 6:214. 10.1186/s40168-018-0609-y 30514368 PMC6280456

[B50] Sterndorff E. B. Russel J. Jakobsen J. Mortensen M. S. Gori K. Herschend J. (2020). The T-shirt microbiome is distinct between individuals and shaped by washing and fabric type. *Environ. Res.* 185:109449. 10.1016/j.envres.2020.109449 32278157

[B51] Sugita T. Yamazaki T. Makimura K. Cho O. Yamada S. Ohshima H. (2016). Comprehensive analysis of the skin fungal microbiota of astronauts during a half-year stay at the International Space Station. *Sabouraudia* 54 232–239. 10.1093/mmy/myv121 26773135

[B52] Szostak-Kotowa J. (2004). Biodeterioration of textiles. *Intern. Biodeteriorat. Biodegrad.* 53 165–170. 10.1016/S0964-8305(03)00090-8

[B53] Taylor D. L. Bruns T. D. (1999). Community structure of ectomycorrhizal fungi in a *Pinus muricata* forest: minimal overlap between the mature forest and resistant propagule communities. *Mol. Ecol.* 8 1837–1850. 10.1046/j.1365-294x.1999.00773.x 10620228

[B54] Truong D. T. Franzosa E. A. Tickle T. L. Scholz M. Weingart G. Pasolli E. (2015). MetaPhlAn2 for enhanced metagenomic taxonomic profiling. *Nat. Methods* 12 902–903. 10.1038/nmeth.3589 26418763

[B55] Turnbaugh P. J. Ley R. E. Hamady M. Fraser-Liggett C. M. Knight R. Gordon J. I. (2007). The human microbiome project. *Nature* 449 804–810. 10.1038/nature06244 17943116 PMC3709439

[B56] Turner S. Pryer K. M. Miao V. P. Palmer J. D. (1999). Investigating deep phylogenetic relationships among cyanobacteria and plastids by small subunit rRNA sequence analysis. *J. Eukaryot. Microbiol.* 46 327–338. 10.1111/j.1550-7408.1999.tb04612.x 10461381

[B57] Vaishampayan P. Probst A. J. La Duc M. T. Bargoma E. Benardini J. N. Andersen G. L. (2013). New perspectives on viable microbial communities in low-biomass cleanroom environments. *ISME J.* 7 312–324. 10.1038/ismej.2012.114 23051695 PMC3554398

[B58] Venkateswaran K. Hattori N. La Duc M. T. Kern R. (2003). ATP as a biomarker of viable microorganisms in clean-room facilities. *J. Microbiol. Methods* 52 367–377. 10.1016/s0167-7012(02)00192-6 12531506

[B59] Watson R. D. (2014). *Modified Advanced Crew Escape Suit Intravehicular Activity Suit for Extravehicular Activity Mobility Evaluations.* Available online at: http://hdl.handle.net/2346/59685

[B60] Webb J. S. Nixon M. Eastwood I. M. Greenhalgh M. Robson G. D. Handley P. S. (2000). Fungal colonization and biodeterioration of plasticized polyvinyl chloride. *Appl. Environ. Microbiol.* 66 3194–3200. 10.1128/aem.66.8.3194-3200.2000 10919769 PMC92133

[B61] Westwood J. Burnett M. Spratt D. Ball M. Wilson D. J. Wellsteed S. (2014). The hospital microbiome project: meeting report for the UK science and innovation network UK-USA workshop ‘beating the superbugs: hospital microbiome studies for tackling antimicrobial resistance’, October 14th 2013. *Stand. Genom. Sci.* 9:12. 10.1186/1944-3277-9-12

