# Supplementary figures and images for: Characterization of Spacesuit Associated Microbial Communities and Their Implications for NASA Missions
Source: Front Microbiol. 2021 Jul 29;12:608478. doi: 10.3389/fmicb.2021.608478 (PMC8358432; doi:10.3389/fmicb.2021.608478)

Supplementary Figure S1

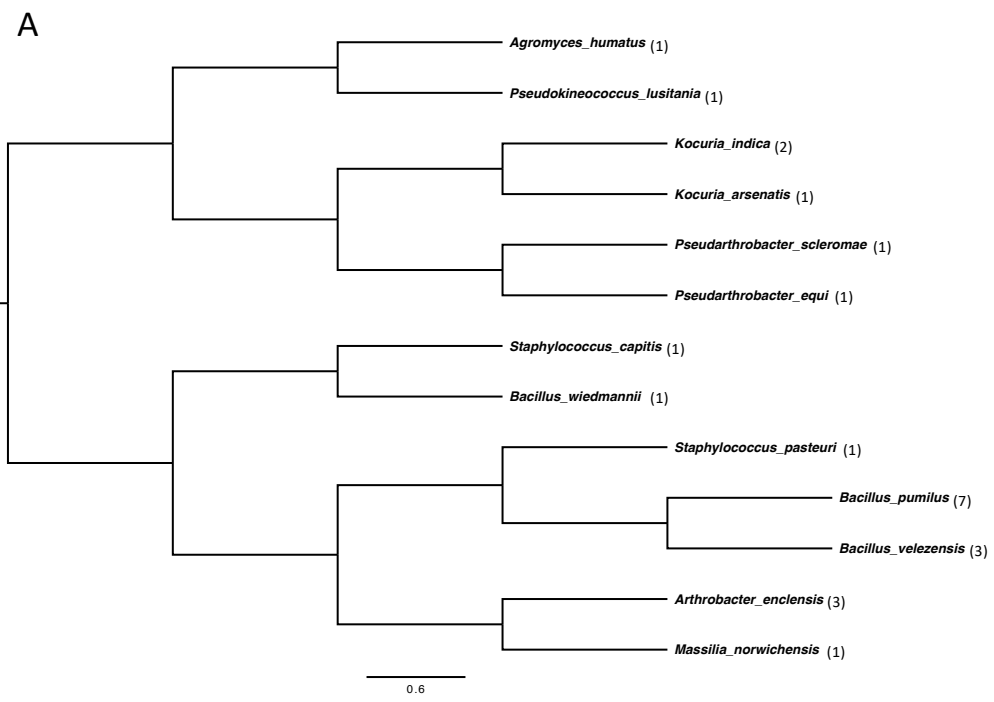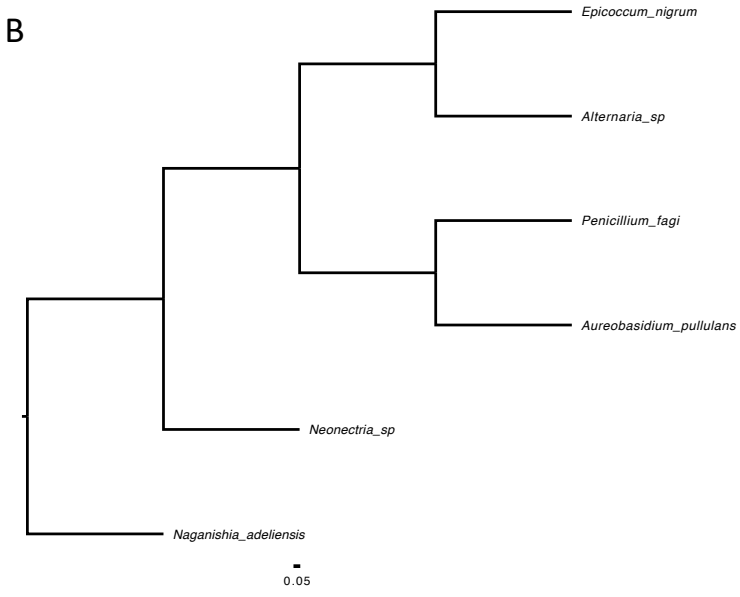

Supplement: Supplementary Figure 1 — Phylogenetic tree of cultivated strains (bacteria [A] and fungi [B]) isolated from spacesuit surfaces. [file Image_1.pdf]

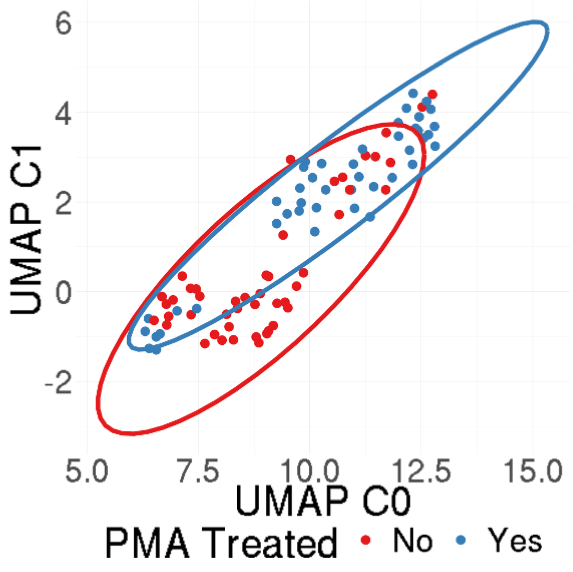

Supplement: Supplementary Figure 2 — UMAP of taxonomic profiles from all samples colored by whether the samples were treated with PMA or not. [file Image_2.tiff]
